# SWIIM study protocol: a pre-post study evaluating the implementation of an established pediatric diabetes management program to improve glycemic outcomes for children and young people with type 1 diabetes

**DOI:** 10.3389/fendo.2026.1750200

**Published:** 2026-03-26

**Authors:** Christopher M. Lawrence, Carmel E. Smart, Megan Paterson, John Wiggers, Anthea Bill, Christopher Oldmeadow, Bruce R. King

**Affiliations:** 1 John Hunter Children’s Hospital, Pediatric Endocrinology and Diabetes, New Lambton Heights, NSW, Australia; 2 Hunter Medical Research Institute, New Lambton Heights, NSW, Australia; 3 School of Medicine and Public Health, College of Health Medicine and Wellbeing, The University of Newcastle, Callaghan, NSW, Australia; 4 Population Health, Hunter New England Local Health District, Wallsend, NSW, Australia

**Keywords:** diabetes, diabetes education, diabetes management, implementation science, pediatric diabetes care

## Abstract

**Background:**

Most young people with type 1 diabetes (T1D) fail to achieve internationally recommended glycemic targets despite recent advances in diabetes technology and education. There is significant variability in management practices and glycemic outcomes across diabetes services, and the need for a more unified approach has been recognized. The Success with Individualized Insulin Management (SWIIM) program, developed at John Hunter Children’s Hospital (JHCH) in New South Wales (NSW), Australia, has demonstrated glycemic outcomes among the best reported nationally for pediatric diabetes. However, its feasibility and effectiveness have not been tested.

**Objective:**

This study aimed to evaluate the implementation of SWIIM across metropolitan and non-metropolitan healthcare settings in NSW, assessing its feasibility, cost-effectiveness, and impact on clinical outcomes.

**Methods:**

A pre–post-implementation study will be conducted in three hospitals: two non-metropolitan sites in the Mid-North Coast Local Health District (MNCLHD) and one metropolitan site in the Nepean Blue Mountains Local Health District (NBMLHD). An education program will be delivered to clinicians with written resources and clinical support. All children and young people aged 0–18 years with T1D receiving care at these sites receive SWIIM for diabetes management. Data will be collected over an 18-month implementation period with retrospective pre-intervention data for comparison. The primary outcome was the change in HbA1c levels over the implementation period. Secondary outcomes included continuous glucose monitoring (CGM) metrics, insulin therapy modality, adherence to complication screening, hospitalization rates, and program feasibility indicators. Acceptability will be assessed using validated patient- and clinician-reported measures. A cost-effectiveness analysis will be performed to determine the financial impact of implementing the SWIIM over standard care.

**Discussion:**

This study aims to evaluate the implementation of a structured, evidence-based pediatric diabetes management program in diverse Australian health service contexts. If successful, SWIIM could provide a scalable and sustainable model of care that improves outcomes and healthcare delivery for pediatric T1D.

## Background

Type 1 diabetes (T1D) can result in significant morbidity and mortality if not optimally managed. The Diabetes Control and Complication Trial (DCCT) of young people and adults demonstrated that over a 30 year follow up period intensive insulin management coupled with comprehensive diabetes education reduced the rate of diabetes-related complications by 30%–49% and lowered mortality by 33% (1). The widespread uptake of diabetes technologies (continuous glucose monitoring [CGM] and insulin pump therapy [IPT]) and the availability of automated insulin pump systems over the 20 years since the initial DCCT publication have been linked to improved glycemic control and improved patient and carer quality of life (2, 3). Consequently, various guidelines, including those of the International Society for Pediatric and Adolescent Diabetes (ISPAD) (4) and the National Institute for Health and Care Excellence (NICE) (5), recommend an HbA1c target of 6.5% (48 mmol/mol) where there is access to advanced diabetes technologies to minimize the risk of diabetes-related complications.

Despite these advances and guidelines, most children and young people with T1D worldwide do not meet the recommended target. The T1D Exchange reported that only 17% of youth in their large database met the target of 7.5% (58 mmol/mol) between 2016 and 2018 (6). In 2022, it was reported that only 12.5% of adolescents and young adults with T1D in Australia achieved the recommended HbA1c target of 7.0% (7). Importantly, there is significant variability in outcomes between diabetes services, which can be attributed to various patient-, clinician-, and system-level factors (8, 9). There is a need for improved evidence-based management strategies that enable young people and their families to achieve these recommendations.

The Success with Individualized Insulin Management (SWIIM) Program is a pediatric diabetes management program encompassing inpatient and outpatient education and management delivered by a multidisciplinary team at the John Hunter Children’s Hospital (JHCH), a tertiary center in NSW, Australia, since 2004. Using the SWIIM program, the JHCH consistently achieves some of the best outcomes for pediatric T1D in Australia and globally (10). The outcomes of the SWIIM and details of the program have been published (11–13). The effectiveness and feasibility of implementing of SWIIM at other sites have not been tested in a research environment.

Approximately 27% of the Australian population lives in non-metropolitan areas (14) and faces unique geographical, financial, and health system challenges, resulting in poorer health outcomes than those living in metropolitan areas. The Modified Monash Model (15) (**Supplementary Table 1**) classifies areas from metropolitan to remote based on the population size and geographic remoteness. In non-metropolitan areas, the lack of consistent reporting of outcome data means that the true burden of T1D is not fully understood (16). The limited existing literature suggests that people with T1D in non-metropolitan areas are less likely to meet glycemic targets (16, 17), have less access to specialist healthcare resources (18), and experience higher psychological burden (19) than their metropolitan counterparts. Additionally, there is no standardized model of care for delivering diabetes care tailored to the non-metropolitan healthcare delivery context.

When access to specialist care is available through outreach clinics, young people with diabetes report satisfaction with their healthcare (20). However, this model can result in fragmented access to services, and when specialist care is unavailable, there is increased reliance on primary healthcare providers who may not have extensive experience with T1D (20).With rapidly advancing technology becoming part of routine T1D care, there is potential for widening health inequity due to lack of access to specialized knowledge and education. A model of care that utilizes existing resources, upskills local providers, and empowers families living with T1D may be a more sustainable model for delivering recommended care and resulting in improved health equity across non-metropolitan areas. Such a model aligns with the proposed service delivery strategy of the Australian National Rural Health Alliance to upskill and support local clinicians and reduce reliance on fly-in–fly-out models (21).

The education and empowerment of children and young people with diabetes and their families are vital for effective diabetes treatment (22). The ISPAD recommends that structured, individualized education be delivered by a multidisciplinary team and provides guidance on the key aspects of diabetes education (23). The complexity of day-to-day management and the paucity of evidence-based structured diabetes management programs for the pediatric population means that there is significant diversity in the educational practices delivered by diabetes teams globally (24).

This study aimed to determine whether the implementation of SWIIM results in improved diabetes outcomes and patient and clinician satisfaction over standard care in metropolitan and non-metropolitan healthcare settings. Ethics approval was granted by the Hunter New England Health Research Ethics Committee (approval no. 2022/ETH01116).

## Methods

### Study design and setting

A pre-post implementation study will be conducted at three sites providing outpatient and inpatient pediatric diabetes care in New South Wales (NSW), Australia. Two of the sites (Port Macquarie and Coffs Harbour) are level 5 public hospitals in the Mid-North Coast Local Health District (MNCLHD). The third center (Nepean) was a level 6 tertiary public hospital in the metropolitan Nepean Blue Mountains Local Health District (NBMLHD). The characteristics of the health districts in comparison to the JHCH, where SWIIM was developed, are presented in **Supplementary Table 2**. The sites were selected to allow analysis of SWIIM implementation in a variety of settings with differing staffing levels and diabetes-specific expertise across metropolitan and non-metropolitan populations.

The implementation of a practice change intervention will occur over an 18-month period (**Supplementary F****igure 1**), during which eligible patients will be recruited. The intervention will be delivered simultaneously across the three sites. Given that the intervention occurs at a site-wide level, a within-center randomization protocol would not be possible. Pre-intervention (usual care) data will be retrospectively obtained from the electronic medical records of recruited the patients. Patients who are newly diagnosed during the study period or for whom retrospective data are not available will also be included for statistical analysis at the available time points (see *Statistical analysis*).

#### Patient involvement

Patients attending the JHCH pediatric diabetes clinic were heavily involved in the development of SWIIM since its inception in 2004. Patients were not directly involved in the study design or methodology; however, Aboriginal community feedback from the study sites was sought in the design of the research project.

### Participants and recruitment

#### Pediatric diabetes services

In the MNCLHD, where there are 110 young people with type 1 diabetes, the average HbA1c level is 8.6%. Each of the two sites is served by a general pediatrician, diabetes educator, and dietitian. The diabetes educator and dietitian were also shared with adult diabetes services. The inpatient ward at each site is a mixed pediatric unit staffed by pediatric trained nurses, and there is access to social workers and Aboriginal health officers that service the entire hospital. Pediatric endocrinologists from JHCH provide six monthly outreach clinics to MNCLHD, an arrangement that began 24 months prior to the study commencement. Patients are reviewed in outpatient clinics three to four times per year.

In NBMLHD, where there are 220 young people with T1D, the average HbA1c level is 8.1%. The team comprises pediatric endocrinologists, dietitians, diabetes educators, and social workers. Patients are reviewed in outpatient clinics three to four times per year. The inpatient ward is a mixed pediatric unit staffed by pediatric trained nurses and there is access to Aboriginal health officers.

#### Clinicians

The practice change intervention will be delivered to all clinicians providing care for young people with type 1 diabetes at the three sites: diabetes nurse educators, diabetes dietitians, social workers, ward nurses and dietitians, Aboriginal health workers, medical practitioners (pediatricians/endocrinologists, fellows, registrars, resident medical officers), and students. At the completion of the intervention period, clinicians who attended SWIIM education sessions and provided a contact email address will be invited by email to complete the digital clinician satisfaction survey and participate in a semi-structured interview.

#### Young people with type 1 diabetes

All young people aged 0–18 years with an existing diagnosis of type 1 diabetes or a new diagnosis within the study period in the catchment area of the three study sites will receive SWIIM education and management from local teams as part of routine care. Patients and their accompanying carers will be recruited by local clinicians for data collection as part of the study at their first face-to-face contact point during the intervention period. Written consent will be obtained to complete the satisfaction questionnaires and collect clinical data as part of the study. Patients and carers will have the opportunity to decline participation in the study or withdraw their consent at any point. Participation in the study did not alter the patient’s receipt of SWIIM as part of clinical care given the practice-wide intervention.

### Intervention

#### SWIIM model of care

The Success with Individualized Insulin Management (SWIIM) Program is an award-winning, patient-centered, multi-component model of care developed and implemented at the JHCH since 2004 (11). It comprises the management of type 1 diabetes from diagnosis to transition to adult services and is delivered by a multidisciplinary team. Patient management under SWIIM begins with inpatient admission for the initiation of ongoing flexible insulin dosing and diabetes education. SWIIM management principles were incorporated into all outpatient reviews. The SWIIM management principles include:

1. Teamwork: The team will operate using the principles of Collaboration, Openness, Respect and Empowerment. All members of the diabetes team provided consistent messaging to families. Team members collaborate to reinforce the messages of other team members.
2. Standardized clinic targets for glycemic metrics: Standardized targets for glucose levels (GLs), Hba1c and CGM metrics will be agreed upon and communicated by the team. A target GL of 3.5 mmol/L–8.0 mmol/L is recommended for all ages at all times of the day. An HbA1c target of 6.5% is recommended for all ages.
3. Insulin dosing and adjustment: Insulin is administered 15 min before four meals per day (breakfast, lunch, afternoon tea, and dinner) for all ages for MDI use and before all meals for IPT. Individualized insulin dosing calculations were provided for each meal and adjusted at regular intervals.
4. Structured eating and mealtime routine: A structured approach to eating, informed by the Australian Dietary Guidelines 2013 (25) is advised, with three main meals and two snack times per day. Individualized recommendations for the maximum/minimum carbohydrate amounts per meal and snack were provided. Additional snacking between meals and at bedtime is discouraged.
5. Exercise management: Physical activity is recommended based on the Australian physical activity and exercise guidelines (26), and advice for insulin adjustment for exercise is provided.
6. Carbohydrate counting from diagnosis: Carbohydrate counting is initiated with the first dose of insulin at the diagnosis of T1D. Education on carbohydrate counting is provided in accordance with ISPAD guidelines during the initial inpatient admission (23).
7. Supervision of insulin administration: Carers and educators are encouraged to supervise all doses of insulin.
8. Continuous glucose monitoring: CGM is commenced as soon as possible after diagnosis, and all young people are encouraged to use CGM for at least 90% of the time. Families were educated on CGM interpretation in accordance with the ISPAD guidelines (27) and encouraged to review CGM patterns at regular intervals.
9. Family education and empowerment: From initial admission, families receive education in decision-making regarding insulin adjustment, management of exercise, and sick days. Following discharge from the hospital, daily phone contact with educators was maintained to provide further directed training on insulin adjustment based on GLs. Written sick-day plans provide guidance on insulin adjustment and when to seek medical attention.
10. Identification and management of psychological comorbidities: Communication within families is encouraged to maintain good psychological health. If families express concerns about psychological health, a brief intervention with a social worker is arranged, and/or a recommendation for GP review is made for consideration of mental health intervention.

The model of care will be delivered to families with the following components:

1. Inpatient insulin initiation and education program for newly diagnosed type 1 diabetes: A standardized education package for newly diagnosed type 1 diabetes was delivered by diabetes nurse educators, dietitians, and social workers over a 5–7-day inpatient admission. Medical officers initiate and adjust subcutaneous insulin doses. Receipt of education is verified by an education checklist completed by families and educators and a carbohydrate counting quiz performed prior to discharge. Following discharge, daily phone follow-ups for ongoing education and insulin dose adjustment were provided for 7–10 days.
2. Three monthly outpatient follow-ups: Young people will be reviewed by a diabetes educator and dietitian 2 weeks after diagnosis. Thereafter, young people are reviewed in outpatient clinics four times per year by physicians. At least one review each with the diabetes educator, dietitian, and social worker (where available) is arranged annually. During outpatient appointments, insulin doses are titrated, and SWIIM management principles are communicated to patients and families.
3. School education: Schools, preschools, and daycare centers that each patient attended received educational session delivered by a diabetes educator. An individualized school plan was provided for each patient. Schools can contact diabetes educators by phone during business hours. The school visit is repeated if there is a change in therapy or the child attends a new school.
4. Written resources: A printed 10 Essential Habits document summarizing key SWIIM principles will be provided to all families from the initial inpatient admission or at the first outpatient clinic review during implementation. Additional printed resources will be provided to the local teams and distributed by clinicians at their discretion, including written and pictorial dietary guidance, insulin dosing guidance, and exercise management strategies. Pro forma for individualized written plans for schools, day care centers, and camps will be provided to local teams.
5. Insulin pump therapy initiation: Patients and families who wish to commence insulin pump therapy will be enrolled in an outpatient education program comprising a 1-hour group pre-pump education workshop with a diabetes educator and dietitian, followed by a 1-hour face-to-face session with the educator and pump company representative for commencement of the pump. Daily phone follow-up was provided for the week after pump initiation to adjust insulin doses until euglycemia was achieved. Insulin pump therapy is not commenced until at least 3–6 months after diagnosis to allow for maximal familiarization with MDI administration.
6. Stabilization admissions: At the team’s discretion, elective admissions to the hospital are utilized to repeat the inpatient education program and adjust insulin doses.
7. Hospital staff education: A hospital procedure document on insulin administration, hypoglycemia management, mealtime routine, and glycemic target is available in hard copy on pediatric wards and on the hospital intranet. Thirty-minute face-to-face training sessions with ward nursing staff are delivered by diabetes educators or dietitians at 3–4 monthly intervals, or as requested by ward staff. Dietitians oversee carbohydrate counting of hospital meals to allow for accurate insulin dosing in hospitals. A written dietary guide, individualized to the child’s age, was provided to the nursing staff to inform them of the appropriate carbohydrate quantities for each meal and snack.

### Implementation intervention

A multi-component clinical practice change intervention will be implemented at each site over an 18-month phase to support embedding SWIIM into the participating sites. Clinicians will be supported to provide the SWIIM program as part of the routine inpatient and outpatient management of T1D in children and adolescents. Through collaborative review with JHCH and implementation site clinicians, aspects of both inpatient and outpatient delivery of SWIIM may be adjusted to suit the local context and the available resources. Deviations from the standard SWIIM implementation will be reported in the final analysis.

#### Implementation intervention strategy development

The following strategies were undertaken to develop implementation strategies:

1. Collaborative project development meetings were held with key stakeholders at the participating sites to identify barriers and enablers to the implementation of the SWIIM program.
2. The priority barriers were mapped to the Theoretical Domains Framework (TDF) techniques for behavior change (28).
3. Consultation with Aboriginal health workers at the participating sites was undertaken to ensure that the implementation was culturally appropriate.
4. The final refinement of the implementation strategies was undertaken following consultation with key clinicians and managers across the participating health districts.

#### Implementation intervention strategies

The implementation strategies used to support the introduction of SWIIM within the three study sites are presented in **Supplementary Table 3**. The TDF domains that formed the basis for each strategy, as well as the barriers that the strategy intended to address, are listed with a description of each strategy.

### Data collection procedures and measures

#### Patient characteristics

At recruitment (which occurs during the intervention period), the research staff will extract demographic data from the electronic medical records, including age, sex, duration of diabetes, and Aboriginal/Torres Strait Islander status. Area-level socioeconomic status will be assigned using residential postcodes mapped to SEIFA’s Index of Relative Socio-economic Disadvantage (reported in quintiles) (29).

#### Primary outcome measures

The primary outcome was glycemic control, measured as HbA1c (%) for each patient, obtained from the EMR. Analyses will be conducted on individual-level data, with modelled effects expressed as changes in the monthly clinic mean HbA1c from the 6-month pre-implementation period to the end of the 18-month implementation period (**Supplementary F****igure 1**). Additional primary outcome data from MNCLHD sites will be collected for the 12 months prior to the commencement of JHCH pediatric endocrinologist outreach clinics through to the commencement of implementation.

#### Secondary clinical outcome measures

Additional patient clinical outcome data were obtained from the electronic medical records at the MNCLHD and NBMLHD sites throughout the pre-intervention and implementation periods. The collected data included:

1. Body mass index (BMI) and BMI standard deviation.
2. Method of insulin administration (fixed insulin dose regimes, flexible multiple daily injections, insulin pump therapy).
3. CGM metrics: Sensor wear time, time in range (3.9 mmol/L–10.0 mmol/L, 70 mg/dL–180 mg/dL), time in tight range (3.9 mmol/L–7.8 mmol/L), time below range (<3.9 mmol/L, <70 mg/dL), time in hypoglycemia, level 2 (<3.0 mmol/L, <54 mg/dL), time above range (>10 mmol/L, >180 mg/dL), and time in hyperglycemia, level 2 (>13.9 mmol/L, >250 mg/dL).
4. Adherence to international recommendations for diabetes-related complication screening (30) in the 12 months preceding implementation to study completion.
5. Number of hospitalizations for diabetes in the 12 months preceding implementation to study completion (not including initial diagnosis).

#### Feasibility of program implementation

Feasibility will be assessed by patient-reported receipt of diabetes-related education as part of the SWIIM program using a diabetes education checklist. The written diabetes education checklist is completed during all inpatient admissions for new diagnoses of T1D by the diabetes educator, dietitian, and family to acknowledge receipt of diabetes-related education. Adherence to follow-up will be assessed by the number of outpatient episodes with the physician, diabetes educator, and dietitian per year. The Australian type 1 diabetes guideline recommendations will be used as targets (three to four outpatient reviews per year and at least one comprehensive review per year with a diabetes educator and dietitian) (31). Checklist completion data and outpatient clinic attendance will be collected from electronic medical records.

#### End-user acceptability of care

At the initial outpatient visit, clinic staff will administer the Diabetes Treatment Satisfaction Questionnaire (DTSQ) and Problem Area in Diabetes (PAID) Scale to patients with a duration of diabetes of at least 12 months. Participants will have the opportunity to complete this on a digital tablet, paper form, or their own digital device. The DTSQ is a validated survey for rating satisfaction with diabetes treatment and can be repeated to assess changes in satisfaction over time following a new intervention (32). Parent and adolescent versions will be administered as part of the study. The PAID is a widely used measure of diabetes-related distress with demonstrated validity and reliability for the 8–12 (child) and 12–18 year (teen) age ranges (33, 34). Questionnaires will be completed by accompanying carers and children and young people aged 8 years and over at six monthly time points to assess changes in treatment satisfaction and diabetes-related distress following the implementation of SWIIM.

#### Clinician acceptability of care

An electronic multiple-choice questionnaire and semi-structured interview will be conducted at two time points (commencement of implementation and study completion) with the diabetes team clinicians. A SWIIM program-specific 9-item questionnaire was developed by the research staff to rate satisfaction and confidence with diabetes management, team cohesion, and identify barriers and enablers to SWIIM implementation. Changes in the rated satisfaction from the questionnaire will be reported. Thematic analysis will be conducted on the interviews to develop a conceptual model of the key enablers and barriers to SWIIM implementation.

#### Cost effectiveness analysis

The value for money of SWIIM will be addressed in a cost-effectiveness analysis (CEA) of the study cohort to determine whether SWIIM is more cost-effective than usual care in reducing the clinical average HbA1c for young people living with type 1 diabetes, where usual care and intervention periods are defined by pre-post comparison at each site. Economic results will be reported as incremental cost-effectiveness ratios. A sensitivity analysis will be conducted to explore the robustness of the results to uncertainties around key parameters.

### Data collection and storage

All data will be stored in a REDCap database administered by the Hunter Medical Research Institute and retained for 15 years. Any potentially identifiable information obtained for this research project will be treated as confidential and securely stored. Information will be coded and de-identified as necessary. Data will be available only to primary researchers and statisticians.

### Power and sample size calculation

Based on the analysis of longitudinal JHCH clinical data, the within-patient variability for HbA1c is low (standard deviation of 0.6% over a 12-month period). To detect a change in HbA1c of 1% at a significance level of 0.05 with power 0.9, the sample size across the three sites needed is 25. If the within-patient variability at the three sites was double that of JHCH (standard deviation of 1.2%), the required sample size would be 90. Allowing for a loss-to-follow-up of 10%, we would aim to recruit a minimum of 100 patients at baseline. There are currently 330 patients across the three sites, and recruitment will continue throughout the study for newly diagnosed patients, with a potential sample size of approximately 400 at study close. This would allow for a recruitment rate of 33% to ensure that the sample size is met.

### Statistical analysis

The primary analysis will evaluate the change in glycemic control, measured by HbA1c at the individual level, from the 6-month pre-implementation baseline period to the end of the 18-month implementation period. Data will be modeled at the patient level, with clinic means over time used as the primary summary measure for interpretation.

A linear mixed-effects segmented regression model will be fitted, with fixed effects for time pre-intervention (monthly index), period (0 = per-implementation, 1 = implementation), time post intervention (which is zero during pre-intervention months and increments monthly thereafter), site, and patient-level covariates: age, sex, diabetes duration, Aboriginal and Torres Strait Islander status, and SEIFA quintile. The model will include a random intercept for patients to account for potential repeated measurements, and an autoregressive (AR(1)) correlation structure will be examined to account for within-patient autocorrelation over time. Effects will be expressed as (i) immediate level change and (ii) change in monthly slope, both relative to the counterfactual pre-implementation trends. Robust (sandwich) standard errors were calculated. Distributional and other modelling assumptions will be assessed by inspecting residual plots, with transformations or different distributional families (e.g., proportional odds logistic models) considered. Secondary outcomes with repeated measures will be assessed using a similar model with an appropriate distributional family dependent on the outcome type.

To assess the impact of medical outreach clinics in the MNCLHD prior to the study onset, an additional comparison of the primary outcome will be made from the commencement of medical outreach clinics to the commencement of the implementation period.

### Governance

The development and implementation of this project were conducted using embedded researchers within the JHCH and the study sites (35). A committee was established for governance oversight of the project, including primary researchers, implementation site champions, and key stakeholders from the Ministry of Health, Aboriginal health, and local health districts. Six monthly governance meetings will be conducted throughout the study period to monitor data collection, review the implementation, and refine implementation strategies. Project teams consisting of JHCH and study site clinicians will operationalize the implementation of SWIIM through the establishment of ongoing local education and guideline development according to the study protocol.

## Discussion

There is a recognized need for improved access to evidence-based best practice care for children and adolescents with T1D in Australia and internationally. The disparity in outcomes reported between centers in Australia and globally indicates a knowledge gap regarding options for optimizing T1D education delivery and management strategies to achieve internationally recommended targets.

To our knowledge, this is the first prospective study that addresses this knowledge gap in both metropolitan and non-metropolitan settings. The pre-post study design is feasible for completion within non-metropolitan sites with limited resources. The study protocol was developed with researchers embedded within the clinical team and with the support of key stakeholders in local health districts, maximizing the chance that clinically relevant changes will be reliably detected and measured.

A limitation of this study is the lack of a contemporaneous control group for comparison. As the intervention is practice-wide, randomization of participants to a within-center control group is not possible. There is a lack of publicly available benchmarking data to provide a comparison group for individuals who do not attend the enrolled centers. Therefore, the causal attribution of the observed changes must be interpreted with caution.

The implementation strategies in this study were developed according to established implementation science frameworks and using feedback from clinicians involved in diabetes care. If positive changes in clinical and operational outcomes are demonstrated, this study will provide evidence to support the implementation of SWIIM in other services and health districts. This study may also provide a framework for implementing other management programs in non-metropolitan areas.
