# Supplementary material for: SWIIM study protocol: a pre-post study evaluating the implementation of an established pediatric diabetes management program to improve glycemic outcomes for children and young people with type 1 diabetes
Source: Front Endocrinol (Lausanne). 2026 Mar 26;17:1750200. doi: 10.3389/fendo.2026.1750200 (PMC13062189; doi:10.3389/fendo.2026.1750200)
Supplement: Supplementary file 1 [file Table1.docx]

|  | | | | | | | | | | | | |  |
| --- | --- | --- | --- | --- | --- | --- | --- | --- | --- | --- | --- | --- | --- |
|  | **Pre-implementation** | | | | | | **Implementation** | | | | **Post-implementation** | |  |
|  |  | | | | | |  | | | |  | |  |
| **MNCLHD** |  |  | JHCH Paediatric endocrinologist outreach clinics | | | | SWIIM implementation | | | |  |  |  |
|  |  |  |  |  |  |  |  |  |  |  |  |  |  |
| *Clinical data collection* |  |  |  |  |  |  |  |  |  |  |  |  |  |
|  |  |  |  |  |  |  |  |  |  |  |  |  |  |
| *Questionnaire data collection* |  |  |  |  |  |  |  |  |  |  |  |  |  |
|  |  |  |  |  |  |  |  |  |  |  |  |  |  |
| **NBMLHD** |  |  |  |  |  |  | SWIIM implementation | | | |  |  |  |
|  |  |  |  |  |  |  |  |  |  |  |  |  |  |
| *Clinical data collection* |  |  |  |  |  |  |  |  |  |  |  |  |  |
|  |  |  |  |  |  |  |  |  |  |  |  |  |  |
| *Questionnaire data collection* |  |  |  |  |  |  |  |  |  |  |  |  |  |
|  |  |  |  |  |  |  |  |  |  |  |  |  |  |
|  |  |  |  |  |  |  |  |  |  |  | Data analysis and manuscript preparation | |  |
|  |  |  |  |  |  |  |  |  |  |  |  |  |  |
|  | **-36** | **-30** | **-24** | **-18** | **-12** | **-6** | **0** | **6** | **12** | **18** | **24** |  |  |
|  | **Months** | | | | | | | | | | | |  |

**Figure 1.** Study timeline. MNCLHD = Mid-North Coast Local Health District, NBMLHD = Nepean Blue Mountains Local Health District

**Table 1.** The Modified Monash Model classification of areas in Australia. Adapted from Department of Health, Disability and Ageing 2024.^16^

| MM 1 | Metropolitan areas |
| --- | --- |
| MM 2 | Regional centres |
| MM 3 | Large rural towns |
| MM 4 | Medium rural towns |
| MM 5 | Small rural towns |
| MM 6 | Remote communities |
| MM 7 | Very remote communities |

**Table 2.** Characteristics of John Hunter Children’s Hospital where SWIIM was developed with the intervention sites as at September 2023. MNCLHD comprises two regional paediatric diabetes services, NBMLHD comprises one urban paediatric diabetes service.
SWIIM = Success With Individualised Insulin Management; JHCH = John Hunter Children’s Hospital; MNCLHD = Mid-North Coast Local Health District; NBMLHD = Nepean Blue Mountains Local Health District; MMM = Modified Monash Model

|  | **SWIIM (JHCH)** | **MNCLHD** | **NBMLHD** |
| --- | --- | --- | --- |
| No. patients in clinic with diagnosis of type 1 diabetes | 388 | 110 | 200 |
| Average HbA1c (IFCC) | 6.9% | 8.6% | 8.1% |
| **Staffing** Paediatric endocrinologist Paediatrician Diabetes nurse educator Dietitian Social worker | ✓ X ✓ ✓ ✓ | X ✓ ✓ ✓ X | ✓ X ✓ ✓ ✓ |
| MMM category of centre | MM1 | MM3 | MM1 |

**Table 3.** Implementation intervention strategies. TDF = Theoretical domains framework

| Implementation strategy | TDF domain/s | Identified barriers | Mapped behaviour change technique | Strategy description |
| --- | --- | --- | --- | --- |
| 1. Leadership and managerial supervision^34^ | Professional role  Belief about consequences | Clinicians perceive that adoption of a new management program for paediatric diabetes would not be supported by their managers. | Social processes of encouragement, pressure, support  Persuasive communication | Hospital executive and clinical leaders have endorsed the project and agreed to support its implementation. |
| 2. Local service champions^34^ | Social/professional role and identity  Motivation and goals  Social influences | Clinician belief that barriers within the local health system limit the ability to enact change in diabetes management  Clinician belief that families of young people with type 1 diabetes will not accept a change in diabetes management | Social processes of encouragement, pressure, support  Persuasive communication  Modelling/demonstration of behaviour by others  Self monitoring | A dietitian and diabetes educator will champion the implementation at each site. They will train staff, monitor rollout and provide feedback to clinicians. |
| 3. Educational meetings and clinician support^35,36^ | Knowledge  Skills  Social/professional role and identity  Beliefs about capabilities  Environmental context and resources | Clinicians perceive that change in clinician behaviour will be difficult to achieve  Clinician belief that families of young people with type 1 diabetes will not accept a change in diabetes management | Planning/implementation  Social processes of encouragement, pressure, support  Modelling/demonstration of behaviour by others | Study site clinicians will visit JHCH to observe/receive education on SWIIM.  A 2 hour face-to-face education session for diabetes team members and hospital staff will be provided prior to program implementation.  JHCH clinicians will virtually meet with study site champions weekly for the first month after the initial educational visit, then monthly for the remainder of the study period. Study site clinicians can contact JHCH clinicians by phone for support during business hours.  A second face-to-face site visit will occur at the 6 month time point.  An introductory education package will be provided for new team members/ward staff that are onboarded following the initial education visit. |
| 4. Educational materials^35^ | Knowledge  Skills  Environmental context and resources  Beliefs about capabilities  Memory, attention, decision, processes | Clinician lack of knowledge in the procedure for insulin adjustment using individualised insulin dosing cards  Clinician belief that families of young people with type 1 diabetes will not accept a change in diabetes management | Planning, implementation  Prompts, triggers, cues  Environmental changes | SWIIM program documents, insulin dosing cards and educational materials will be provided to study sites. Study site clinicians will be supported to establish local education and diabetes team meetings, and develop local clinical practice guidelines for adoption within the local health district. Aspects of inpatient and outpatient management may be adjusted to suit the local context. |
| 5. Audit and feedback^37^ | Social influences  Beliefs about capabilities  Environmental context and resources | Manager belief that additional burden will be placed on clinicians to attend education  Clinician belief that management as part of the SWIIM program is more time consuming than standard care | Feedback  Rewards; incentives (including self-evaluation) | A 6 monthly collaborative review meeting will be held to assess progress of implementation, refine implementation strategies and monitor data collection. 6 monthly primary outcome data (clinic average HbA1c) will be provided to clinicians within the study sites for the duration of the study period. |
